# From trial to clinic: psilocybin’s efficacy in routine treatment-resistant depression

**DOI:** 10.1016/j.lanepe.2026.101780

**Published:** 2026-07-15

**Authors:** Vassilis Martiadis

**Affiliations:** ASL Napoli 1 Centro, Department of Mental Health, Naples, Italy

The question of whether the striking antidepressant effects of psilocybin reported in randomised trials can be reproduced in everyday care remains largely unanswered. This is because those trials enrol highly selected patients, withdraw concomitant medication and deliver treatment under tightly standardised protocols. In this issue, Jungwirth et al. provide one of the first responses from routine clinical practice.^1^ Among 19 adults with treatment-resistant depression (TRD) who were treated with psilocybin under Switzerland’s limited medical use framework, clinician-rated symptoms decreased substantially (a Montgomery–Åsberg reduction of approximately 11 points; Hedges’ g ≈ 1.4). However, response and remission rates of 33% and 22%, respectively, on the MADRS were much lower than those reported in landmark efficacy trials.^2^^,^^3^ It is the difference between these two figures, rather than either number alone, that is the key message (Fig. 1).

**Fig. 1:**
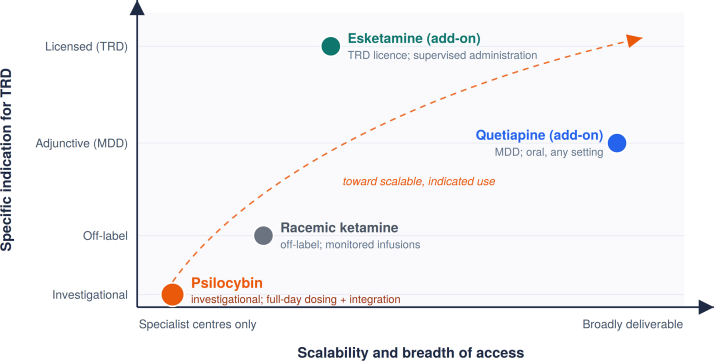
**The treatment-resistant depression therapeutic landscape.** Selected options positioned by the strength of their specific regulatory indication for TRD (vertical axis) and the scalability of their delivery model (horizontal axis). Esketamine and quetiapine are both add-ons to an oral antidepressant, but esketamine carries a TRD-specific licence and requires supervised administration, whereas quetiapine is indicated for major depression rather than treatment resistance; racemic ketamine remains off-label; psilocybin shows promising efficacy yet the lowest scalability. The dashed arrow indicates the trajectory—toward both a regulatory indication and scalable delivery—that psilocybin would need to follow.

Treatment-resistant depression remains one of the most pressing unmet needs in psychiatry, and treatments with a specific regulatory indication are few and far between. Esketamine nasal spray is essentially the sole TRD-specific option; it is licensed for TRD, though only as an add-on to an oral antidepressant. Quetiapine is the main adjunctive alternative, but it is indicated for major depression rather than treatment resistance. Racemic ketamine is used off-label, and national guidelines diverge on what to recommend and how to appraise the evidence.^4^ Against this fragmented backdrop, a smaller effect in routine care is instructive for the clinician rather than disappointing. The efficacy obtained under experimental conditions rarely transfers intact to the clinic. Psychiatry learned this when only about a third of a broadly representative group of outpatients responded to a first guideline-based antidepressant in STAR∗D.^5^ The Zurich cohort embodies precisely the patients that trials exclude: those who have tried multiple antidepressants, have comorbidities, have experienced chronic episodes and are taking concomitant medication rather than having stopped taking it. Yet, a shortfall is not inevitable. Despite a comparably complex real-world population, esketamine has demonstrated effectiveness that is broadly consistent with that observed in its registration trials. Response and remission accrue over the first months of treatment.^6^ Whether psilocybin’s real-world effectiveness will likewise converge with trial estimates or whether the observed discrepancy will persist is the first open question.

The study’s setting raises a second question that it does not itself address: scalability. This was not a private clinic, but a tertiary academic hospital. Even there, the process was extraordinarily resource-intensive, involving full-day supervised dosing, several preparation and integration sessions, and multiple specially trained therapists for each patient.^7^ The fact that only 19 patients could be assembled over roughly two years illustrates the bottleneck. TRD affects a large and growing population across Europe. If effective psychedelic therapy remains available only in a few ultra-specialised centres, access rather than efficacy will be the main limiting factor in its impact. The problem is compounded by equity, because such centres cluster in well-resourced regions and academic hubs, leaving rural and underserved populations furthest from a treatment that is positioned as a last resort. A therapy that cannot be delivered on the scale and across the geography of the disorder it targets cannot, by itself, narrow the TRD treatment gap.

Several further questions lie beyond the scope of the study and should be addressed in the next phase. The first of these is durability: as outcomes were captured at the earliest post-dose assessment, the persistence of benefit, the property that TRD treatment most needs, remains untested. Relatedly, the authors’ sparse session-wise data give no clear indication that repeated dosing adds benefit, leaving the optimal number and spacing of sessions unresolved. Thirdly, the study reveals little about the function of the treatment, such as quality of life, employment and social recovery, which ultimately determine its worth. The fourth issue, which is the most consequential for policy, concerns cost-effectiveness and capacity: who should be trained, where, at what cost, and how can equitable access be ensured across systems with divergent regulation and reimbursement? Identifying predictors of sustained response would enable costly interventions to be targeted at those most likely to benefit, thereby improving both outcomes and economic viability. European compassionate-use and regulated-access programmes are already expanding unevenly,^8^ and without harmonised indications, outcome measures and shared registries, the field risks accumulating heterogeneous case series rather than comparable, poolable evidence.

None of this diminishes the contribution. Jungwirth and colleagues demonstrate that rigorous, real-world observation of psilocybin is feasible and that some of its potential may survive in the context of complex, routine care. The more challenging task, shared by the wider TRD field and psychedelics research, is to demonstrate that a treatment not only works, but also improves lives durably and can be delivered fairly and at scale. Until these three factors are pursued together, positive results will remain confined to the centres able to generate them.

## Contributors

VM is the sole author. VM conceived and wrote the Comment, critically revised it and approved the final version; VM is responsible for the figure and accepts full responsibility for the content and the decision to submit for publication.

## Declaration of use of artificial intelligence (AI)

The author used an AI-based assistant (Claude [Claude Opus 4.8], Anthropic) to support the language editing and formatting of this Commentary, under the author’s direction. The topic, scientific content, interpretation, and argumentation reflect the author’s own expertise; the author reviewed, revised, and source-verified all AI-assisted text, and selected and verified all cited references.

## Declaration of interests

VM has received advisory board and/or speaker honoraria from Johnson & Johnson, Lundbeck, Otsuka, Teva, and Angelini.
